# Biosignatures of Exposure/Transmission and Immunity

**DOI:** 10.4269/ajtmh.15-0037

**Published:** 2015-09-02

**Authors:** Christopher L. King, D. Huw Davies, Phil Felgner, Elizabeth Baum, Aarti Jain, Arlo Randall, Kevin Tetteh, Christopher J. Drakeley, Bryan Greenhouse

**Affiliations:** Center for Global Health and Diseases and Veterans Affairs Medical Center, Cleveland, Ohio; Division of Infectious Diseases, Department of Medicine, University of California, Irvine, Irvine, California; Antigen Discovery Inc., Irvine, California; Faculty of Infectious and Tropical Diseases, London School of Tropical Medicine and Hygiene, London, United Kingdom; Division of Infectious Diseases, Department of Medicine, University of California, San Francisco, San Francisco, California

## Abstract

A blood test that captures cumulative exposure over time and assesses levels of naturally acquired immunity (NAI) would provide a critical tool to monitor the impact of interventions to reduce malaria transmission and broaden our understanding of how NAI develops around the world as a function of age and exposure. This article describes a collaborative effort in multiple International Centers of Excellence in Malaria Research (ICEMRs) to develop such tests using malaria-specific antibody responses as biosignatures of transmission and immunity. The focus is on the use of *Plasmodium falciparum* and *Plasmodium vivax* protein microarrays to identify a panel of the most informative antibody responses in diverse malaria-endemic settings representing an unparalleled spectrum of malaria transmission and malaria species mixes before and after interventions to reduce malaria transmission.

## Introduction

The ability to accurately and rapidly monitor the effectiveness of malaria interventions to reduce transmission and the burden of disease is essential for an effective control program. Current methods usually measure the prevalence of parasitemia in children, and either passive or active case detection of symptomatic malaria. Although these methods are useful, they can be resource intensive and imprecise, especially in areas with low or changing transmission. A blood test that captures cumulative exposure, recent exposure, and assesses levels of naturally acquired immunity (NAI) against malaria would be extremely valuable. This article describes a collaborative effort in multiple International Centers of Excellence in Malaria Research (ICEMRs) to develop such a test using malaria-specific antibody responses as biosignatures of transmission and immunity. The rationale of using antibodies comes from the observation that antibodies against specific parasite antigens persist in time and at reasonably stable concentrations, even when transmission is seasonal. The antibodies in sera are relatively easy to measure through experiments using basic techniques such as enzyme-linked immunosorbent assay or more advanced approaches such as bead arrays or protein microarrays on a genomic scale.

Human infection with *Plasmodium* spp. induces potent antibody responses that increase with subsequent exposure and include three basic characteristics: 1) circulating immunoglobulin, especially immunoglobulin G (IgG); 2) plasma cells, predominantly located in the bone marrow, that produce these antibodies; and 3) memory B cells that can rapidly expand to generate additional antibody-producing plasma cells, more memory B cells, and potentially immunoregulatory B cells. These three components are often correlated, but sometimes may not be.^1^,^2^ For example, the absence or low levels of serum antibodies may belie the presence of good memory responses or the reverse.

**Figure 1.**
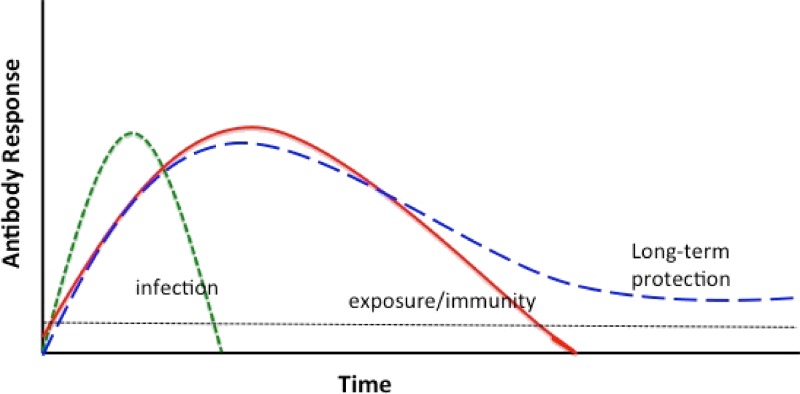
Preferable characteristics of antibody responses to antigens for biosignatures of exposure and immunity. Under conditions of moderate-to-low transmission antibody responses last months may be optimal (solid line). By contrast, antibody responses that last weeks (dotted line) might be better measure of recent infection or perhaps exposure under high transmission conditions. Horizontal dotted line indicates threshold of detection.

Immunological memory to malaria as measured by the presence of serum antibodies to plasmodia blood-stage antigens can persist for years as can the presence of circulating memory B cells,^3^–^6^ even in the absence of malaria exposure.^3^ As with most infections, antibody levels to plasmodia wane in the absence of reinfection and increase with reexposure.^7^ The durability of antibody responses to malaria antigens can be highly variable. The mechanisms underlying this variability are unclear,^8^–^10^ but are likely related to the way the antigens are processed and presented by individuals (i.e., the host genetic component), the type of memory T-cell response induced, whether the memory B cells and/or plasma cells are expanded, maintained, or destroyed, and/or whether antibodies are consumed in the process of removing malaria parasites.^11^ These varied kinetics of antibody responses provide a large and diverse set of potential biosignatures of exposure and acquired immunity, the ideal characteristics of which will vary depending on the epidemiologic setting. An ideal antibody response to monitor transmission or NAI might be one that persists several months in moderate-to-low transmission conditions. By contrast, antibodies with shorter half-life might be preferable in high-transmission settings (Figure 1

).

The strength of cross-ICEMR studies is the ability to use standardized assays to measure antibody responses in a broad variety of epidemiological settings ranging from areas with high transmission to those with low or unstable transmission. Moreover, many of the ICEMR sites are undergoing malaria control measures such that the effects of the reduction in malaria transmission on infection and disease can be evaluated. This study describes the development of a *Plasmodium falciparum* (*Pf*)/*Plasmodium vivax* (*Pv*) protein microarray designed to measure biosignatures of transmission and acquired immunity across a large number of malaria-endemic sites. Protein microarrays of the format used in the cross-ICEMR survey have been previously used to measure antibody responses to malaria antigens in the context of exposure and acquired immunity.^12^–^18^ This study is novel in that a standard platform is used across many sites that span the breadth of malaria transmission and comprise areas with exclusively *Pf*, exclusively *Pv* and locations where both parasites are transmitted.

## Methods

### Study sites.

A detailed discussion of the ICEMR study sites and epidemiology related to these sites are indicated in other articles in this volume. An overview of the studies and related epidemiology and the number of individuals whose sera was used to probe the arrays is provided in Table 1.

### Ethical approvals.

Ethical approval was obtained from all ICEMR centers in the United States and the FWA-accredited ethical review boards in the respective malaria endemic areas.

### Protein microarray platform.

All sequences for this particular array were derived from a single reference genome (3D7). This approach allowed the evaluation of a greater breadth of antigens, but limited evaluation of the effect of antigenic variation on the antibody response. Separate studies outside the ICEMR program to evaluate the latter are ongoing. Microarrays (Antigen Discovery Inc., Irvine, CA) were printed and probed following previously published methods.^12^,^19^ In brief, proteins were expressed in cell-free in vitro transcription/translation (IVTT) reactions from T7 promoter plasmids in our existing *Pf* clone library. Each protein contains N-terminal poly-His and C-terminal hemagglutinin tags for quality control. Serum samples were diluted 1:200 in Protein Array Blocking buffer (GVS Life Sciences, Sanford, ME) supplemented with 10% *Escherichia coli* lysate (Antigen Discovery Inc., Irvine, CA), and incubated overnight on arrays at 4°C. After incubation with secondary antibody and wash steps, slides were scanned on a GenePix 4200AL (Molecular Devices, Sunnyvale, CA) and spot intensities quantified using ScanArray Express software (Perkin Elmer, Waltham, MA).

### Analysis of the array data.

The mean pixel intensity for the spot was subtracted by the local background around the spot and a floor of 100 was set for the data. The IVTT protein spot intensities were normalized either by dividing by or subtracting the multiple IVTT control spots (IVTT reactions lacking plasmid template) that are printed throughout the chip. Statistical analyses were performed on the base-2 logarithm of the normalized data using the base stats package in R (www.r-project.org) to calculate two-sided *t* test and *P* values adjusted for false discovery. The fold-over control (FOC) normalization reduces variation in signals that could potentially arise between probing operations performed at different times. FOC normalization provides a relative measure of the specific antibody binding to the nonspecific antibody binding to the IVTT controls. Thus, with the normalized, log_2_-transformed data, a value of 0.0 means the intensity is not different from background and a value of 1.0 indicates a doubling with respect to background. However in some analyses, straight mean fluorescence indices (MFI) minus IVTT background were used.

The number of seropositive antigens in different locations was determined relative to North American controls using significance analysis of microarray implemented in the MultiExperiment Viewer microarray software suite from TM4 (www.tm4.org). In this analysis, only positive serological responses that were significantly different from the U.S. controls were counted as seropositive.

### Upload of array data to PlasmoDB.

All data were uploaded on PlasmoDB (http://plasmodb.org/plasmo/) that provides free access to information on *Plasmodium* genomes and individual genes identified for the ICEMR array.

## Results

### Development of the Pf500/Pv500 protein array for trans-ICEMR sampling.

**Figure 2.**
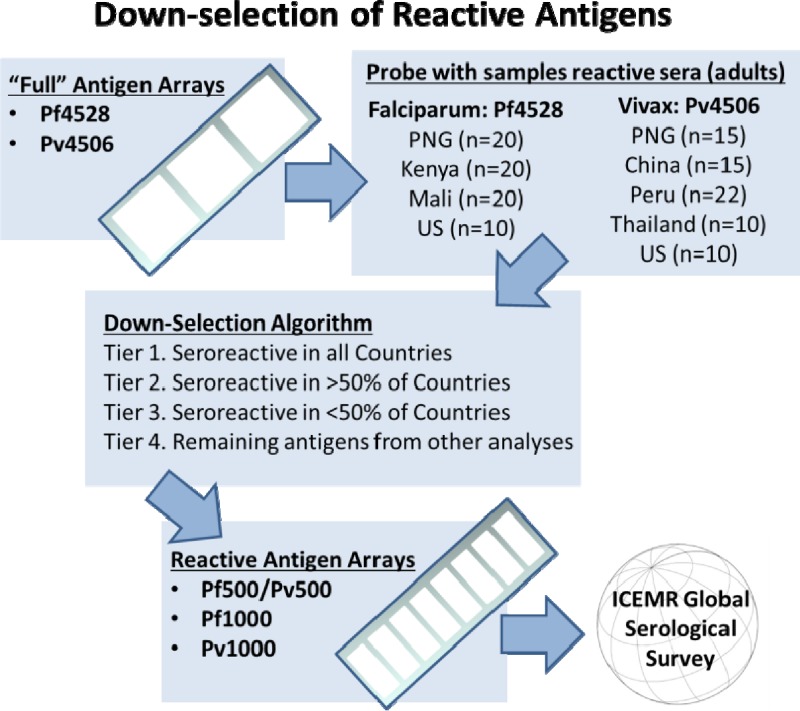
Schematic showing down-selection of antigens for International Centers of Excellence in Malaria Research (ICEMR) Pf500/Pv500 protein microarray.

To develop a protein array that was most informative about exposure and immunity as well as cost effective, we designed an array containing approximately 500 antigens from both *Pf* and *Pv* (Pf500/Pv500) for the trans-ICEMR serological survey. Antigens to be included on this array were down-selected from larger arrays probed with highly reactive sera representative of different populations around the globe. Thus for the *Pf* features, an array of well-known *Pf* genes with 4,528 features (Pf4528) was probed with adult samples from Papua New Guinea (PNG), Kenya, Mali (*N* = 20 each), and the United States as a negative control (*N* = 10). Kenya and Mali are high-transmission areas with ostensibly only *Pf* transmission, while PNG has high transmission of both *Pf* and *Pv* and reacts very strongly with antigens from both species on the protein arrays. For *Pv*, Pv4441^20^ was probed with adult samples from PNG, China (*N* = 15 each), Peru (*N* = 22), Thailand (*N* = 10), and the United States (*N* = 10). Seroreactive antigens in each country were defined as those antigens with an average signal that was above a cutoff defined as the average plus two standard deviations of the U.S. control population. From the compiled list of seroreactive antigens, the top 500 *Pf* and 500 *Pv* antigens were selected using hierarchical filtering. Highest priority was given to antigens seroreactive in all countries tested, with the remaining positions allocated by descending order of reactivity across the countries (Figure 2

). The features selected for the Pf500/Pv500 array were deposited in the NCBI Gene Expression Omnibus database via accession number GPL18316 (www.ncbi.nlm.nih.gov/geo/query/acc.cgi?acc=GPL18316). On the basis of the information from plasmodb.org, the distribution of *Pf* life stage of maximum expression represented on the array were as follows: merozoite 24%, early ring 22%, late schizogony 14%, early trophozoite 14%, early schizogony 11%, late trophozoite 8%, late ring 5%, and null 2%, representing almost exclusively blood-stage antigens.^12^ Life-stage expression information was not available for *P. vivax* proteins.

### Relationship between transmission intensity and breadth of antibody response using samples from the ICEMR surveys.

Figure 3

**Figure 3.**
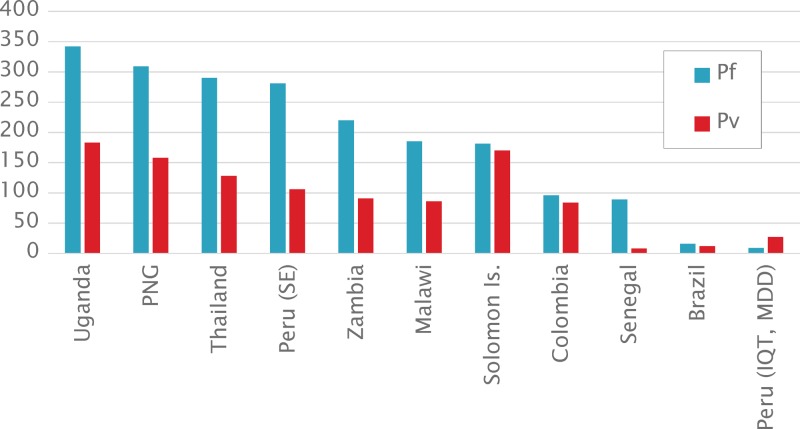
Numbers of different seroreactive antigens detected (significance analysis of microarrays) by samples from different International Centers of Excellence in Malaria Research (ICEMR) regions revealed by probing against the Pf500/Pv500 protein microarray. Table 1 describes the samples tested. PNG = Papua New Guinea; SE = Santa Emilia; MDD = Madre de Dios; IQT = Iquitos.

shows the breadth of response to the array as determined by the number of features recognized. For this analysis, seroreactive adults were used regardless of parasitemic status, with the aim of providing the largest sample sizes. Sera from several sites were highly reactive. For example, of the 500 *Pf* features on the ICEMR array, > 300 were reactive with sera from PNG (Pacific southwest ICEMR). Adults from Uganda (Jinja, Kanungu, and Tororo combined), Thailand (Tak Province), and Santa Emila in Peru detected similar numbers of reactive antigens to that observed in PNG. Overall, sera from sites with higher transmission recognized more antigens in the array compared with areas with lower transmission.

In areas with little or no *Pv* transmission (e.g., Uganda, Malawi, and Zambia), sera recognized many *Pv* features suggesting cross-reaction between antigens (see “Species specificity of assays” below). Similarly in areas with predominantly or only *Pv* transmission (Colombia, Brazil, and Thailand), sera also recognized *Pf* antigens. Overall sera recognized fewer *Pv* than *Pf* features. This may represent higher parasitemia levels associated with *Pf* infections and stronger boosting of antibody responses to blood-stage antigens.

### Species specificity of assays.

**Figure 4.**
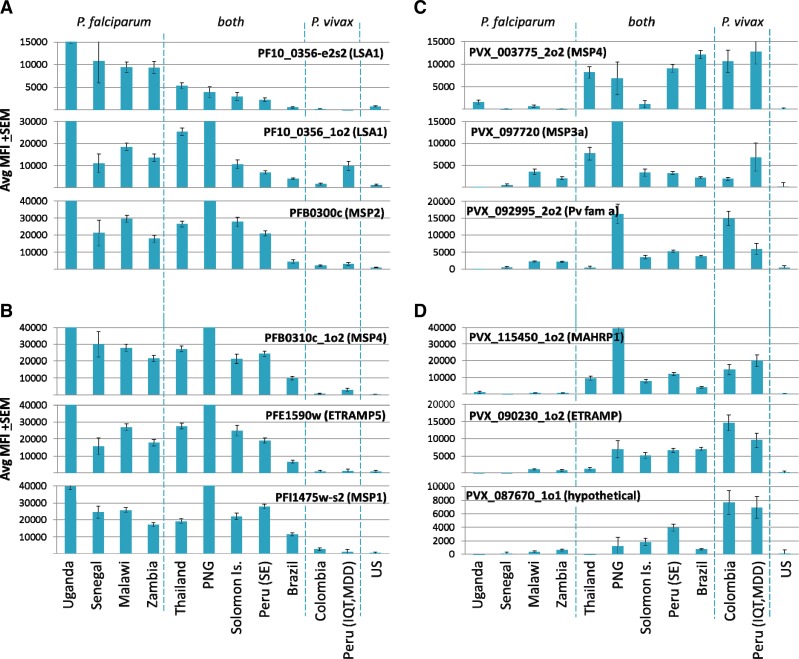
Trans International Centers of Excellence in Malaria Research (ICEMR) seroreactivity patterns of candidate *Plasmodium falciparum*- and *Plasmodium vivax*-specific antigens. Shown are Pf500/Pv500 protein microarray data of representative parasite-specific antigens, identified by comparing *P. falciparum* and *P. vivax* mono-infections from Uganda and Peru, respectively (see text for details). *P. falciparum* antigens, without (**A**) and with (**B**) orthologs in *P. vivax*. *Plasmodium vivax* antigens, without (**C**) and with (**D**) orthologs in *P. falciparum*. Reactivity for each antigen is expressed as mean fluorescence intensity ±standard error of the mean (SEM). Different ICEMRs are listed at the foot of the figure (see Figure 2 for details) and have been sorted from left to right by the mean fluorescence intensity (MFI) for PF10_0356-e2s2 (LSA1). These sites resolved into three distinct groups as indicated by the dashed lines: predominantly *P. falciparum* (left), predominantly *P. vivax* (right), and both species (middle).

Intuitively, *Plasmodium* species-specific genes may give rise to species-specific antigens. However, the majority of genes in *Pf* and *Pv* are orthologs of each other, and therefore, it is likely they share epitopes for antibody recognition. This portends antigenic cross-reactivity that will complicate the interpretation of antibody recognition of malaria proteins, especially in areas of the world where there is coincident exposure to both *Pf* and *Pv*. Through the ICEMR program, it has been possible to assess serology in essentially mono-exposed populations. Thus, the antibody profiles in samples from sub-Saharan Uganda, Senegal, Malawi, and Zambia primarily represent *Pf* mono-infections, although a low level of *Pv* exposure might occur in some of these populations.^21^,^22^ In addition to robust reactivity to *Pf* antigens, there was also strong reactivity to a subset of *Pv* antigens, which we attribute to antigenic cross-reactivity (see Figure 3). Reciprocally, certain populations in Peru have been identified as *Pv* mono-infections (notably a mining community in Madre de Dios where there has not been a case of falciparum malaria for over a decade). Here too there was cross-reactivity to a subset of *Pf* antigens. As might be predicted, many orthologous genes encode cross-reactive antigens and reciprocally non-orthologous antigens are more abundant among those that are species specific. However, there were exceptions as shown in Figure 4

. For example, there are antigens encoded by *Pf* that have shared orthologs in *Pv*, but which were recognized only in *Pf* infections (Figure 4B). Similarly, there are *Pv* antigens that have orthologs in *Pf* but which were *Pv* specific (Figure 4D). Presumably, this phenomenon reflects the differing biology of *Pf* and *Pv* infections that changes the antigenicity of a given homolog depending on the infecting species. In this context, “antigenicity” may be influenced by any number of properties of the protein that determine its exposure to the immune system, such as its abundance, duration of expression, or stability. Other lead candidates for species-specific antigens recognized by antibodies are those for which there are no orthologous antigens between these two species (Figure 4A and C). Such analyses help to identify lead candidates for species-specific biosignatures of transmission and immunity in *Pf* and *Pv* co-endemic areas. It should be mentioned that *Plasmodium malariae* (*Pm*) and/or *Plasmodium ovale* (*Po*) are also co-endemic in some ICEMR study sites^23^,^24^ that may also express cross-reactive antigens to *Pf* and/or *Pv*.

### Identification of antigens most strongly associated with transmission.

**Figure 5.**
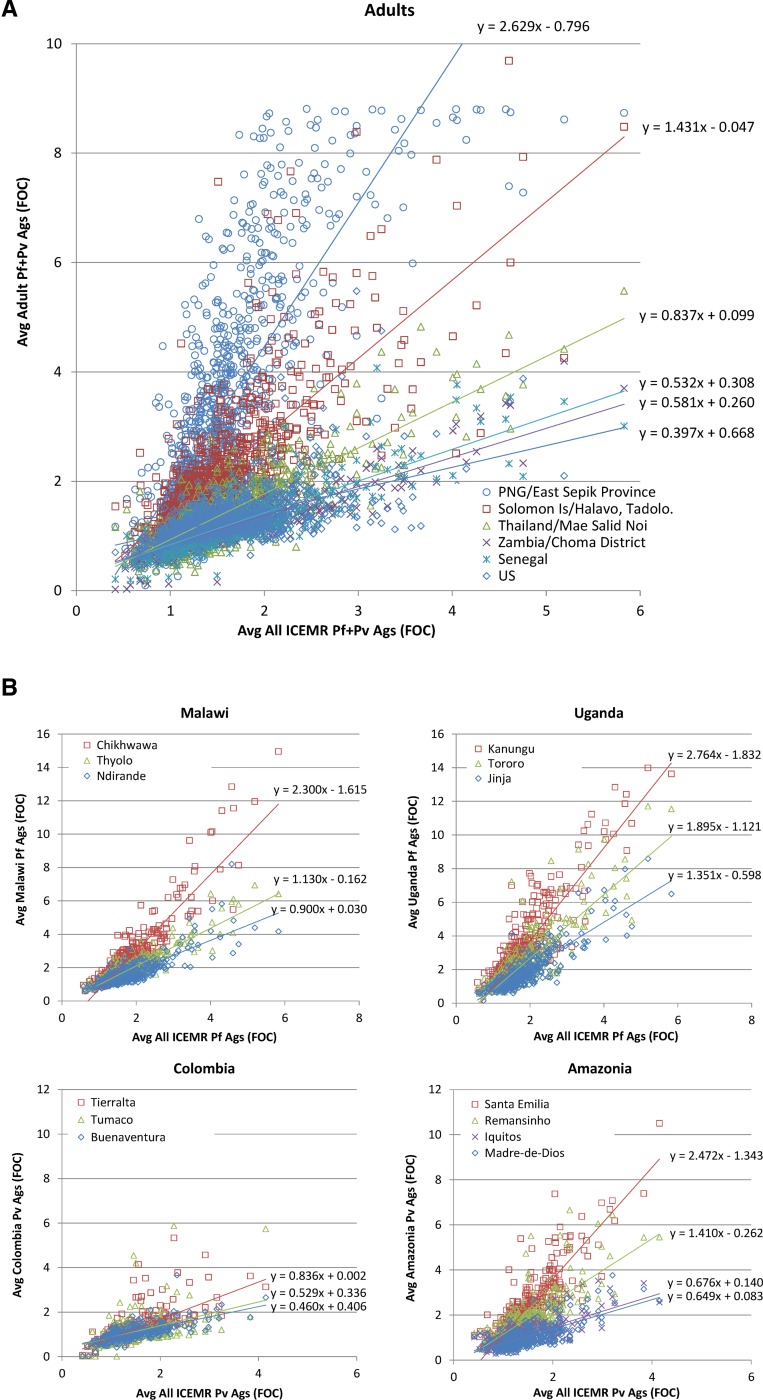
(**A**) A trans International Centers of Excellence in Malaria Research (trans-ICEMR) survey of seroreactivity of adults (≥ 18 years; Table 1) in several malaria endemic regions (PNG, Solomon Islands, Thailand, Senegal, Zambia, and the United States). In each ICEMR site, average signals of adults in each ICEMR site (*y* axis) were plotted against the average for all ICEMR sites (*x* axis). Each point represents the mean fold over control (FOC) signal intensity for all individuals examined in the particular population to a particular antigen. The number of individuals examined in each area is shown in Table 1. PNG = Papua New Guinea; US = United States. The slope of the line is proportional to the overall intensity of response to both *Pf* and *Pv* protein arrays. (**B**) Shows similar data, but the level of reactivity of multiple locations within four different ICEMR sites, two with predominantly *Pf* (sub-Saharan Africa) and two predominantly *Pv* (South America).

To determine which antibody responses are likely to be most informative in different epidemiologic settings, it is critical to understand the natural variation in responses across these settings. Shown in Figure 5

is an initial trans-ICEMR analysis of protein microarray data from some of the sites sampled as part of the ICMER program for which biosignatures of exposure will be evaluated. The figure provides an overall summary of the breadth and variability of antibody response between different populations with varying transmission.

Figure 5A is derived from cross-sectional studies of adults from different ICEMRs and compared with those of the United States. The slope of the line is proportional to the breadth and intensity of the antibody profile with respect to levels of responses in all the populations examined, with each dot representing a single antigen. The *y* axis value for each antigen represents the mean reactivity to that antigen in the particular population and the *x* axis the average reactivity for all sites. Note that the United States has the lowest slope and provides a useful baseline.

It should be emphasized that an analysis of this kind is highly dependent on the sampled populations, and it is possible to find a range of seroreactivity within the same country, particularly in low-transmission areas, which may be the reason why the slopes of the lines are low (i.e., most antigens have low reactivity) for Senegal and Zambia. For example, Figure 5B shows individual ICEMR sites with multiple locations within each site, each with differing transmission intensities. In this figure, the antigens represented have been selected to represent the dominant species present in each site. Thus, for Malawi and Uganda, only *P. falciparum* antigens are represented, whereas for Colombia and Amazonia only *P. vivax* antigens are represented. The slopes generated by such analyses for all ICEMR sites tested to date are provided in Table 2.

**Figure 6.**
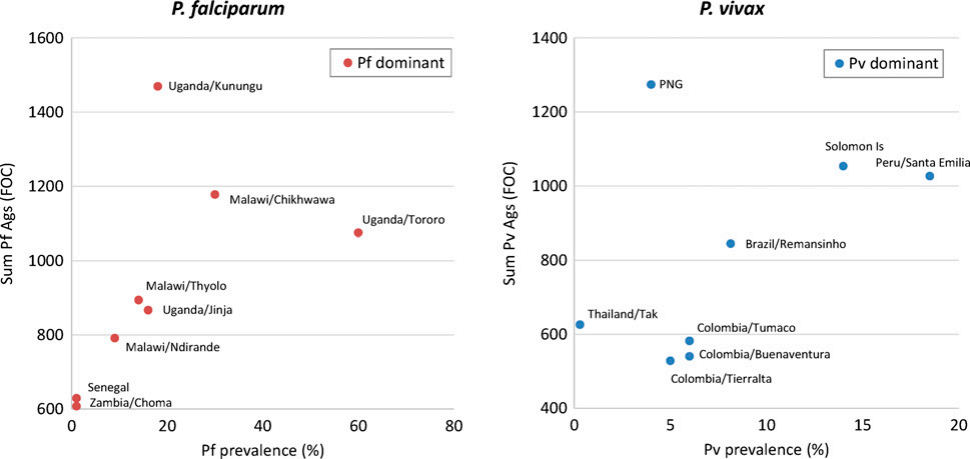
The relationship of parasite prevalence at a population level and the overall breadth and magnitude of serological reactivity as measured by sum log_2_ fold increased over controls (FOC) for every *Pf* antigen on the array. The left panel is *Pf* antigens and sites with predominantly *Pf* transmission. The right panel shows reactivity to *Pv* antigens at International Centers of Excellence in Malaria Research (ICEMR) sites with predominantly *Pv*. Prevalence is based on blood smear and/or rapid diagnostic test (RDT) (see review by Moss W. J., and others in this volume) and may be an underestimate, notably in Thailand.^12^

To better evaluate the relationship of overall seroreactivity in each site with malaria prevalence, the cumulative antibody reactivity to *Pf* antigens for sites that had predominantly falciparum malaria (Figure 6

, left panel) and to *Pv* antigens for areas with primarily vivax malaria (Figure 6, right panel) are shown. The strength and breadth of serological reactivity to *Pf* showed an almost linear correlation between *Pf* prevalence, especially for the three sites in Malawi (Figure 6, left panel). Tororo, Uganda, with the highest prevalence of any of the sites tested, appeared to be an outlier since the overall reactivity was not as high as predicted by the trend. This suggests a complex relationship between exposure and antibody responses. Under low-transmission conditions, a malaria infection can result in lower although persistent antibody responses. Whereas under very high transmission conditions, it is possible that humoral immunity may be dampened by potent immunoregulatory mechanisms, for example, due to rapid consumption of antibodies and impairment of memory in the setting of recurrent, high-density parasitemia.

The correlation between prevalence and *Pv* reactivity also shows the same trend as *Pf*, although the relationship is probably more complex (Figure 6, right panel). First, most sites with predominant *Pv* infections also have some *Pf* transmission that can contribute significant serological cross-reactivity between many *Pv* and *Pf* antigens (Figures 3 and 4). Also, a majority of blood-stage *Pv* infections likely arise from relapsing *Pv* parasites from hypnozoites. The relapsing patterns can differ significantly between sites^25^ and may often present as low-density infections that can be missed by routine surveillance methods including molecular methods. Of note, the Thailand site has documented a significant prevalence of submicroscopic infections (~9% by polymerase chain reaction [PCR]) accounting for its higher than expected serological reactivity.

### Biosignatures of NAI.

Several studies are underway as part of the ICEMR program to define biosignatures of NAI. Three study designs are currently being used to identify individuals with clinical immunity to malaria. It is hypothesized immune subjects will differentially recognize a subset of malaria proteins that would represent a biosignature of clinical immunity. The first and most powerful is the longitudinal cohort study, where individuals are actively and passively followed at regular intervals (often every 2 weeks or monthly) with the aim that most or all malaria infections and illnesses are captured. Biosignatures of protection are measured in serum or plasma samples and are related to risk of clinical malaria, accounting for varied exposure, over the course of follow-up. Such studies are labor intensive and require populations with sufficiently high transmission levels such that enough clinical events are captured to have sufficient statistical power. An example of this approach using the microarray and correlates of immunity (non-ICEMR) was recently published.^26^ Similar studies in the Uganda and southwest Pacific ICEMRs are currently underway.

**Figure 7.**
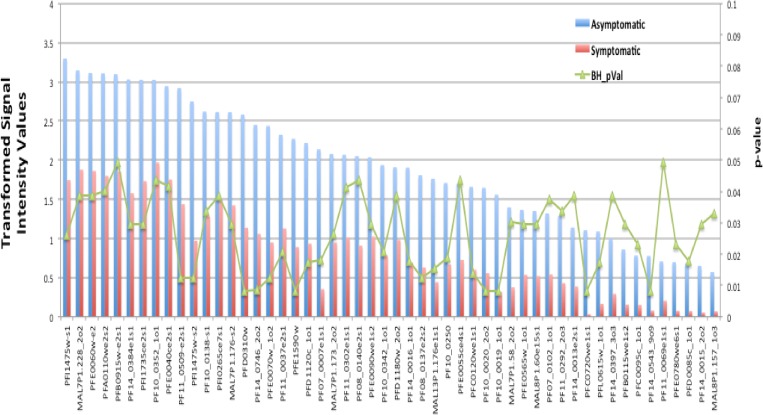
Differential expression of antibodies to 52 malaria antigens in asymptomatic (blue bars) and symptomatic subjects (red bars). Shown are the log-transformed MFI values and the Benjamini–Hochberg corrected *P* values < 0.05.

The second are serial cross-sectional studies in the same population. Often the same individuals are observed repeatedly, but surveys are less frequent than cohort studies and missing data on individuals is much more common than in the cohort studies (there is no active follow-up of individuals). With good passive surveillance, most symptomatic malaria cases are captured. The dynamics of asymptomatic infections are missed however. An example of such a study (also non-ICEMR) showed that children with higher overall serological reactivity to *Pf* antigens on the microarray were significantly less likely to acquire symptomatic malaria compared with those with lower overall serological responses during the transmission season.^13^ Investigators are performing similar analyses with focus on *Pv* in Remansinho, Brazil ICEMR.^27^ The third approach is a comparison of asymptomatic versus symptomatic parasitemic individuals (i.e., a case–control study design) in cross-sectional surveys. Persons with blood-stage malaria infections (often detected by molecular methods such as PCR) in the absence of clinical symptoms are considered to be clinically immune compared with those with symptomatic malaria. Although this approach is the most tractable study design, it has the weakness that an accurate malaria history may not be available, and putatively immune individuals may have been recently ill with malaria. It is also possible that these are just long-standing infections to which the individual has developed some level of strain-specific immunity from prolonged submicroscopic infection. Such studies are underway at a number of sites. An example of such an approach was recently completed in Peru ICEMR.^28^ In this study, sera from symptomatic (*N* = 24) by passive case detection and asymptomatic (*N* = 14) *P. falciparum* malaria infections (blood smear positive in the absence of any malaria referable symptoms in previous 2 weeks) in carefully age-matched (nested) cohorts were probed on the Pf824 microarray to identify differentially recognized antigens that may be associated with protection. The number of prior malaria episodes was similar between the two groups as well as the median age (27 and 32 years for symptomatic versus asymptomatic, respectively). Median parasite densities, however, were more than log-fold higher in symptomatic individuals compared with asymptomatic individuals, which may have contributed to consumption of antibodies. On the basis of mean MFI, 52 antigens were found to be differently reactive between samples from these two different patient groups (*P* value ≤ 0.05, corrected for false discovery Figure 7

). Importantly, the asymptomatic participants had overall higher antibody reactivity. Of these differentially recognized antigens, 13 were identified as conserved *Plasmodium* spp. proteins of unknown function. PfMSP-1 (gene identification, PFI1475w), an established vaccine candidate, showed the highest reactivity among the differentially reactive antigens. Other vaccine candidates found were EBA175 (MAL7P1.176) and liver-stage antigen 3 (LSA3, ID PFB0915w). Ring erythrocyte surface antigen (PFA0110) was also differentially reactive; this protein has been reported to be involved in modifying the biophysical properties of infected erythrocytes and the effect seems to be enhanced by increasing temperature to fever levels. Two heat shock proteins, HSP40 (PFE0055ce4) and HSP70 (MAL7P1.228), were differentially recognized. Several other differentially recognized proteins are involved in interactions of infected red blood cells (RBCs) with uninfected RBCs and endothelial cells: ring-exported protein 1 (ID: PFI1735c), early transcribed membrane proteins (E-TRAMPs) 4,5,10.1, and 14.1 (IDs: PFD1120c, PFE1590w, PF10_0019, and PF14_0016).

It should be emphasized that the majority of antigens on the array did not show any difference between the two groups and some of these had the highest overall seroreactivity that could be potential biosignatures of exposure. Examples of highly seroreactive antigens in both groups are PfMSP10, PfRh2, MSP1, and LSA3.

## Discussion

Much of the data presented in this article provide the basis for more detailed studies to identify optimal antigens for biosignatures of transmission and NAI. In the following discussion, we describe these preliminary findings in the broader context of the present understanding and approaches to identify biosignatures of transmission and acquired immunity.

### Biosignatures of transmission.

Antibody responses to *Plasmodium* have been used for decades to assess exposure to infecting parasites and thus characterize transmission. To date, the most standardized and widely validated method for evaluating exposure via serology is the seroconversion rate (SCR), which appears to be informative across a broad range of transmission settings. Typically SCR is calculated from age-specific seroprevalence, with seropositive individuals representing both presently infected and previously exposed individuals depending on the kinetics of antibody responses to the particular antigen.

Calculation of an SCR extends seroprevalence analysis by calculating the rate at which individuals in a population become seropositive. SCR is analogous to the force-of-infection and is derived from the analysis of seroprevalence as function of age.^29^ A curve is fitted to observed age seroprevalence data, most commonly using reverse catalytic models fit by maximum likelihood.^30^ In these models, age of individuals is assumed to be a proxy of time and individuals move between seronegative and seropositive states on malaria exposure and absence of reinfection. This assumption may not be accurate in all populations where people may migrate from malaria nonendemic to endemic areas such as the Brazilian Amazon. SCR is defined as the rate at which seronegative individuals convert to seropositive and the converse (seropositive individuals reverting to a seronegative state) is the seroreversion rate (SRR). SRR is related to antibody decay in the absence of disease exposure and is related to a variety of factors as described above. Other models that use a stacking process to move between several levels of seropositivity and or antibody data as a continuous variable have also been developed.^31^

Several studies have demonstrated the utility of SCR as a malaria epidemiological measure, which can be collected from a variety of survey types (community, school, and health facility) and that shows good agreement with classic measures of malaria transmission intensity such as the entomological inoculation rate.^30^ Other studies have highlighted the unique property of serology in its ability to estimate historical exposure levels and also to demonstrate reductions in exposure to infection-associated interventions.^32^,^33^ A number of ICEMR programs are using or are planning to use SCR to estimate transmission. In Columbia, estimates of SCR using responses to *Pv* circumsporozoite protein accurately stratified risk between the three sites; in Uganda, estimates of SCR using responses to *Pf* apical membrane antigen 1 also accurately stratified risk between the three sites, in contrast with parasite prevalence.^34^

One important question being addressed in the ICEMR program is defining the optimal antigenic targets for evaluating exposure, including how these may vary in different epidemiologic settings, and when evaluated in different age groups. This is important as the dynamics of antibody acquisition and maintenance vary based on exposure intensity, thus the degree to which some serologic markers are informative of exposure will likely vary in these contexts. Moreover, the different kinetic properties of antigens should allow a more refined characterization of the time elapsed since exposure occurred, for example, using antigens with short half-lives to reflect more recent exposure.^7^

In this study, we developed a targeted protein microarray to examine antibody response to both *Pv* and *Pf* antigens with ultimate aim to identify a set of novel serological assays as biosignatures of transmission and NAI using antibodies targeting a suite of particular proteins. The serological assays that identify transmission versus those for acquired immunity may differ, although there may be some overlap.

Overall, the array identified antigens that were generally more reactive in subjects residing in high- versus low-transmission conditions, however this relationship was imprecise and some individuals living in high-transmission areas had lower overall responses compared with those living in lower transmission areas. Since malaria transmission can be highly heterogenous and vary greatly with age, more precise analyses are currently underway to better control for these variables and to better select the more informative antigens.

The issue of cross-reactivity between antigens especially in areas where both *Pf* and *Pv* are endemic is important. This may not be of great significance where one species is much more prevalent compared with the others, for example, *Pf* in sub-Saharan Africa and *Pv* in many areas of Latin America; however, there are many areas where both are co-endemic. We show some initial approaches to identification non-cross-reactive antigens using serum samples from areas that are only *Pf* or *Pv* endemic from careful studies in the ICEMR. As might be expected, non-orthologous gene products are most likely to be non-cross-reactive between *Pf* and *Pv*, although some orthologous genes are cross-reactive. Studies are currently underway to identify the most informative species-specific antigens.

Another important question being addressed is evaluating whether alternative methods for estimating transmission from serology can provide more information from fewer subjects, and/or information not easily obtainable from current methods, in particular, quantifying recent changes in exposure or defining heterogeneity in exposure at a more granular level. Whether the same antigens are associated with clinical immunity as determined by a similar study design or cohort studies in other malaria-endemic settings are a goal of ongoing ICEMR studies.

A separate protein microarray study, targeted specifically at identifying serologic biomarkers of exposure, is testing the hypothesis that individual-level estimates of exposure can be obtained from a limited number of properly chosen antibody responses. This study is leveraging detailed cohort studies from the east African ICEMR to obtain individual-level exposure data, and using flexible, data adaptive models to identify the most informative biosignatures. Data from these ongoing studies suggest that antibody responses to three properly chosen antigens can provide detailed information regarding recent exposure in individuals and can be aggregated to provide accurate estimates of population-level exposure within and between communities. Protein production of these promising antigens is underway, with plans to evaluate the kinetics of these responses and their ability to inform exposure across multiple epidemiologic settings, taking advantage of the rich epidemiologic data and sample bank being collected as part of the ICEMR program. In addition, separate studies to perform novel candidate discovery across individuals from distinct groups of age and exposure settings are planned. Other studies are currently underway to examine biosignatures of exposure to *Pf* in Zambia and Zimbabwe (South Africa ICEMR), biosignatures of *Pv* in Peru, Brazil (Amazonia ICEMR), in PNG and Solomon Islands (south west Pacific ICEMR), and in Thailand and Columbia where transmission levels are very low.

### Biosignatures of NAI.

NAI is now recognized as a major determinant of the incidence and prevalence of malaria infection and disease in endemic areas.^35^–^38^ Generally, NAI influences not only the age-specific incidence and prevalence of *Pf* and *Pv* infection, but also the pathological processes that underlie the clinical manifestations of infection. Improved understanding of the development and maintenance of NAI and the ability to cope with the severe manifestations of malaria are now particularly important, since effective public health interventions that reduce transmission are being deployed. Knowledge of the underlying mechanisms of NAI will also be invaluable in the design and evaluation of additional tools that prevent and treat malaria-related illness, most especially in constructing vaccines that ultimately eliminate the disease. Moreover, the identification of a biosignature of NAI can help determine whether populations are at a high risk of disease with malaria infection and whether this risk changes as interventions successfully reduce transmission.

Anti-parasite and anti-disease immunity are often correlated, but develop at different rates and likely differ at least in part in their underlying mechanisms of action. Indeed, an ongoing analysis of cohort data from the Ugandan and southwest Pacific ICEMRs aims to quantify the relationships between each of these types of immunity with age and exposure and their relationship to each other. In general, low parasitemia levels in an immunologically naive individual will cause disease so the presence of malaria infection in otherwise clinically asymptomatic individuals reflects immunity to disease, suggesting malaria-exposed individuals develop a type of “tolerance” to malaria infection. What comprises this tolerance remains poorly understood, but likely comprises several components. First is acquisition of immunity to components of the malaria parasites that trigger innate inflammatory responses, for example, antibodies that may interfere with pro-inflammatory glycoprotein to Toll-like receptors.^39^,^40^ Second is development of immunoregulatory responses by the host that attenuate the host response to parasite products such as anti-inflammatory cytokines such as interleukin-10, expansion of malaria-specific regulatory T cells,^41^–^43^ and/or B- and T-cell exhaustion.^44^ Ongoing studies in the Uganda and southwest Pacific ICEMRs are examining such immunoregulatory responses in detail.

**Figure 8.**
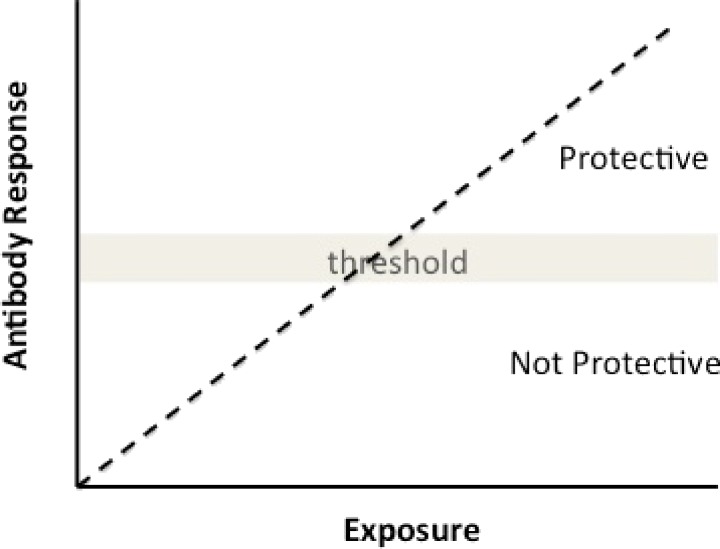
Low antibody levels are predictive of malaria risk, but as antibody levels increase (with increasing exposure) and reach a theoretical threshold, antibodies contribute to protective immunity and serve as biosignatures of malaria immunity.

The mechanisms that govern NAI remain elusive, although our knowledge has expanded greatly over the past several decades. Studies performed over 50 years ago showed that transfer of IgG from clinically immune adults to children symptomatic with malaria markedly attenuated their infection and disease, demonstrating the critical role of antibodies in blood-stage immunity.^45^ To which parasite antigens these protective antibodies bind and how they contribute to clearance of blood-stage parasites has been a focus of intense study. Although absolute in vitro correlates of protection to malaria are unknown, consensus has emerged that there are specific biosignatures of immunity. These include elevated levels of serum antibodies directed at merozoite antigens (particularly invasion ligands) and/or variant surface antigen on infected erythrocytes.^46^–^48^ These responses represent associations rather than causal relations of protective immunity, but they are valuable if they can predict the level of NAI in populations before and after the implementation of malaria control measures. A likely scenario is that once antibody levels that target multiple merozoite antigens involved in erythrocytes invasion and/or surface proteins expressed on the malaria-infected erythrocytes reach a critical threshold, clinical immunity develops (Figure 8

). The decline of these responses below this critical threshold may predict susceptibility to infection.

The overall goal of the trans-ICEMR studies is to identify a constellation of malaria antigen-specific antibodies whose levels are consistently associated with NAI using different epidemiological approaches. However, it may be that the correlates of exposure vary in different epidemiologic settings or age groups. Importantly, as the frequency of clinical malaria diminishes resulting from interventions that reduce malaria transmission, the development of NAI will also diminish or shift to older individuals. As a consequence ideal biosignatures may also change in accordance with epidemiological studies monitoring changes in the incidence and/or prevalence of clinical malaria. The aim is to identify serological tests that can identify individuals lacking adequate NAI resulting in an increased risk of clinical malaria. Such monitoring can alert the public health system when NAI in the population declines below a critical level, leaving the population prone to epidemics with potentially high morbidity. Initial results from the southwest Pacific ICEMR suggest a critical threshold can be defined based on seroreactivity to relatively small group of antigens.^49^

### Additional immunological markers associated with NAI.

Because of its relative simplicity, the primary focus of ICEMR efforts has been on serological biosignatures of transmission and immunity; however, effector mechanisms of immunity are complex. For example, the ability of antibodies to opsonize malaria-infected erythrocytes or merozoites might better correlate with clinical immunity.^50^ Similarly, the ability of antibodies to fix complement and/or block merozoite invasion or schizont release from erythrocytes may identify a clinically important subset of antibodies. Measuring host cellular responses may also be important.^51^ It is likely one or more of these pathways is involved in NAI and may vary with time and parasite strain in the same individual, which has made it difficult to consistently identify robust correlates of immunity.

## Conclusions

The knowledge obtained from ICEMR-related studies will begin to address critical gaps in our understanding of naturally acquired humoral immune response against *Pf* and *Pv* to inform malaria vaccine development and identify potential new biosignatures associated with exposure and protection worldwide. These data will improve and broaden our understanding of how NAI develops around the world as a function of age and exposure and will be useful to monitor eradication and control measures underway in the different ICEMRs. The project may also contribute to the discovery of vaccine antigen candidates to boost NAI to asexual and sexual stage parasites.

## Figures and Tables

**Table 1:** Exposure and ages of samples used for arrays

Study site	Country	No. of samples probed	Exposure category*	Parasites	Ages (years)
Tororo	Uganda	40	High	*Pf*	≥ 18
Jinja	Uganda	40	Moderate	*Pf*	≥ 18
Kanungu	Uganda	40	Moderate	*Pf*	≥ 18
Mugil	Papua New Guinea (PNG)	20	High	*Pf* = *Pv*	≥ 18
Tak	Thailand	153	Moderate	*Pv* > *Pf*	≥ 18
Santa Emilia	Peru	324	Moderate	*Pv* = *Pf*	All ages
Madre de Dios	Peru	40	Low	*Pv*	≥ 18 years
Macha	Zambia	244	Low	*Pf*	All ages
Chikwawa	Malawi	33	High	*Pf*	≥ 18
Thyolo	Malawi	33	Moderate	*Pf*	≥ 18
Ndirande	Malawi	33	Low	*Pf*	≥ 18
Ngella	Solomon Islands	164	Low	*Pv* >> *Pf*	All ages
Tierralta/Cordoba	Columbia	340	Low	*Pv*	All ages
Remansinho	Brazil	281	Moderate	*Pv* >> *Pf*	All ages
	Senegal	160	Low	*Pf*	All ages

* Currently these estimates of exposure are approximate and based on a number of different measures such as parasite incidence and prevalence rates, entomological inoculation rates, and in a few cases the molecular force of infection, which is the number of new malaria infection per year based on molecular typing of parasites.

**Table 2:** Relative antigen reactivity to *Pf* and/or *Pv* arrays across all the ICEMR sites

Country	Location	Slope (all antigens)	Slope (*Pf* antigens)	Slope (*Pv* antigens)
PNG	East Sepik Province	2.629	2.429	2.544
Uganda	Kanungu	2.476	2.764	1.594
Peru	Santa Emilia	2.334	2.248	2.472
Malawi	Chikwawa	2.080	2.300	1.770
Uganda	Tororo	1.671	1.895	1.171
Solomon Islands	Halavo, Tadolo	1.431	1.262	2.048
Uganda	Jinja	1.202	1.351	0.792
Malawi	Thyolo	1.103	1.130	1.147
Brazil	Remansinho	0.935	0.784	1.410
Malawi	Ndirande	0.849	0.900	0.778
Thailand	Mae Salid Noi	0.837	0.833	0.823
Senegal	Senegal	0.581	0.598	0.555
Colombia	Tierralta	0.579	0.487	0.836
Colombia	Tumaco	0.544	0.562	0.529
Zambia	Choma District	0.532	0.545	0.525
Colombia	Buenaventura	0.504	0.532	0.460
Peru	Madre-de-Dios	0.473	0.380	0.649
Peru	Iquitos	0.460	0.363	0.676
United States		0.397	0.322	0.651

ICEMR = International Centers of Excellence in Malaria Research; *Pf* = *Plasmodium falciparum*; *Pv* = *Plasmodium vivax*.
